# Long-term changes of standing balance after lateral ankle sprain using Footscan system: A case report

**DOI:** 10.1097/MD.0000000000036058

**Published:** 2023-11-17

**Authors:** Kwangohk Jun, Hyoshin Eo, Won Mo Koo, Seongho Woo, Jeeihn Lee, Jong Min Kim, Byung Joo Lee, Tae-Woo Nam

**Affiliations:** a Department of Physical Medicine and Rehabilitation, Daegu Fatima Hospital, Dong-gu, Daegu, South Korea; b Department of Physical Medicine and Rehabilitation, Suseong Children’s Hospital, Suseong, Daegu, South Korea; c Department of Physical Medicine and Rehabilitation, Gyeongbuk Regional Rehabilitation Hospital, Gyeongbuk, South Korea.

**Keywords:** ankle instability, Footscan system, lateral ankle sprain, standing balance

## Abstract

**Rationale:**

A lateral ankle sprain (LAS) is a common sports related injury. Ankle instability and balance impairment after injury are common. This case report describes the longitudinal changes in static balance after LAS.

**Patient concerns:**

A 36-year-old man visited our hospital with LAS of the right ankle that occurred during an exercise session. The patient complained of severe pain and swelling of the ankle. The patient was unable to walk a short distance.

**Diagnoses:**

Ultrasound examination showed swelling of the surrounding soft tissues and a partial tear of the right anterior talofibular ligament. In the Doppler scan, vascularity increased around anterior talofibular ligament. No fractures were observed on computed tomography.

**Interventions:**

The patient received analgesics for pain control. The rest, ice, compression, elevation protocol was used. The injured area was protected with a controlled ankle movement walking boot for 2 weeks. Standing balance was measured at 3, 4, 8, 12, and 24 weeks after injury using Footscan.

**Outcome:**

He was able to walk approximately 2 weeks after the injury with reduced pain over time. It was observed that the standing balance improved over time.

**Lesson:**

In this case, it was objectively confirmed that standing balance was restored naturally after LAS.

## 1. Introduction

Acute ankle sprain is a common musculoskeletal injury among athletes. It can be divided into lateral, medial and high/syndesmotic ankle sprains according to the location of the lesion. Lateral ankle sprain (LAS), accounting for about 73% of these, are one of the most common musculoskeletal injuries associated with exercise.^[1]^ One meta-analysis study reported that athletes had a prevalence of 0.93 LAS per 1000 matches or exercises.^[2]^ LAS is caused by hypersupination of the ankle. The most commonly involved ligaments are the anterior talofibular ligament and calcaneofibular ligament.^[3]^ The diagnosis of LAS is usually diagnosed based on the patient’s medical history. However, in severe cases, complications such as fracture or rupture of the ligament, may occur. Therefore, additional radiography, ultrasonography, or magnetic resonance imaging may be performed according to the Ottawa ankle rules.^[4]^ LAS have been shown to recur in 11.9% of cases,^[5]^ and may lead to pain that persists after the acute phase and decreased physical ability. After repeated LAS, in approximately 70% of cases, it may lead to a long-term disability called chronic ankle instability.^[6–8]^ It can also induce osteoarthritis in the long run.^[9,10]^

Chronic ankle instability due to LAS causes laxity and mechanical instability, which can make standing balance unstable.^[11]^ Few quantitative studies have been conducted regarding this matter. In this study, quantitative measurement of long-term changes in static standing balance after LAS was performed using a plantar force measuring plate called the Footscan system.

## 2. Case presentation

**Figure 1.**
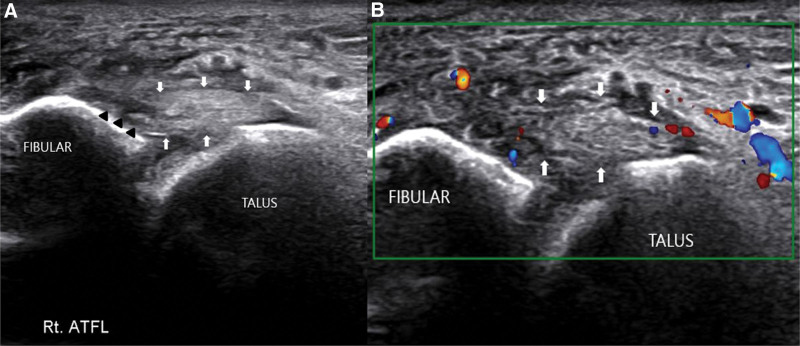
Ultrasonography of right ATFL (arrow). (A) Partial tear of anterior right ATFL (arrowhead). (B) Increased vascularity around right ATFL on doppler image. ATFL = anterior talofibular ligament.

An amateur tennis player, height 177 cm, weight 75 kg, 36 years old male, stepped on a tennis ball with his right foot and sprained his ankle. His right ankle had abrupt and excessive plantarflexion and inversion. Swelling, pain and tenderness were observed on the lateral side of the ankle. After the injury, the patient was unable to walk for more than 4 steps. No fracture was identified on computed tomography. Ultrasonography revealed a partial tear in the right anterior talofibular ligament and increased vascularity of the surrounding soft tissues (Fig. 1).

**Figure 2.**
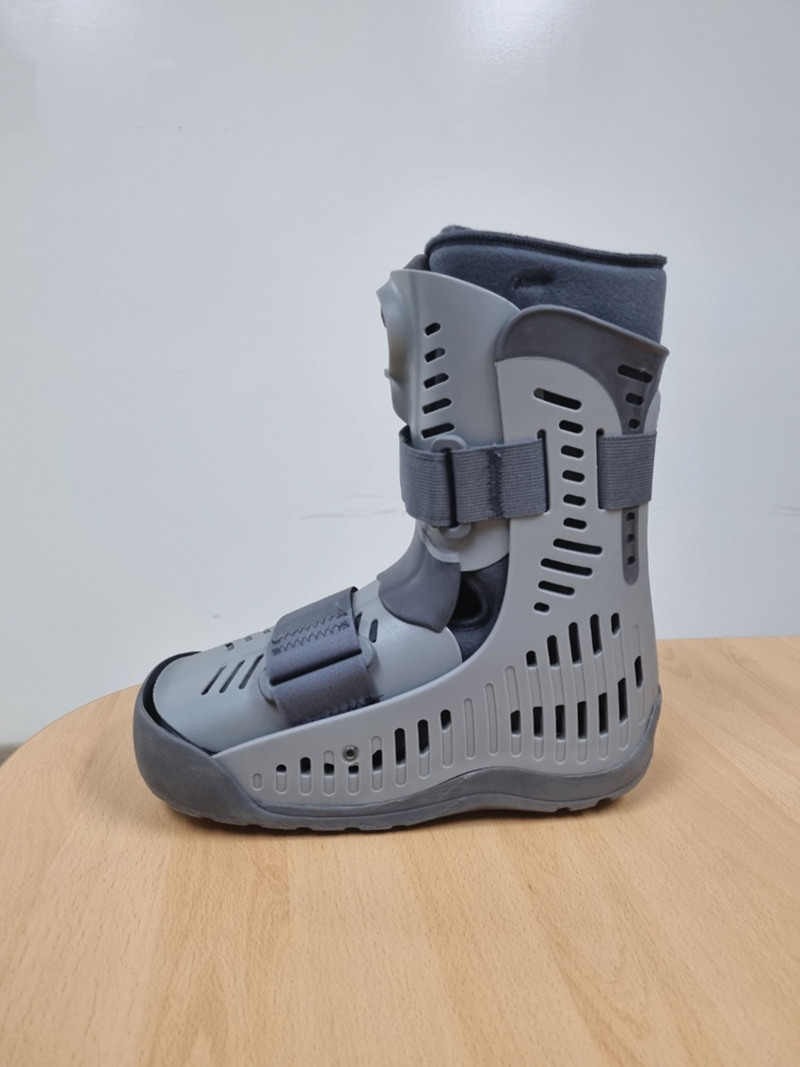
CAM walking boot. CAM = controlled ankle movement.

Based on the symptoms and ultrasound findings, the patent was diagnosed with grade II ankle sprain.^[12]^ He received analgesics for pain control and the rest, ice, compression, elevation protocol.^[13]^ A controlled ankle movement walking boot was applied for 2 weeks (Fig. 2). After 2 weeks, his pain and swelling reduced and he could walk independently in an antalgic gait pattern without a controlled ankle movement walking brace. The patient did not participate in any rehabilitation exercise program. Ankle sprain did not recur during the follow-up period.

**Figure 3.**
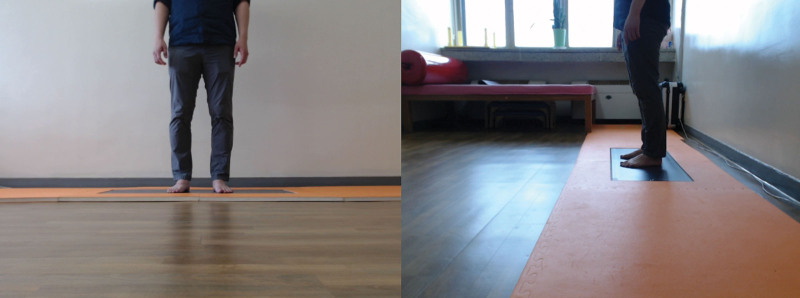
Plantar force measuring plate called Footscan.

**Figure 4.**
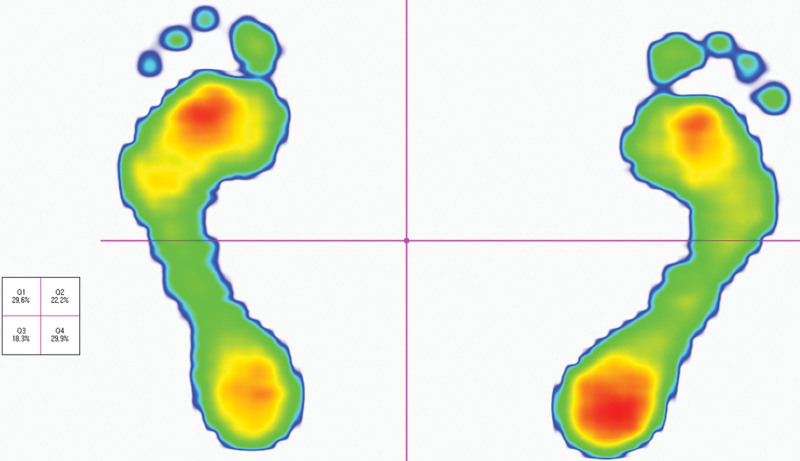
The weight distribution by four quadrants.

Periodic follow-up examinations were performed to measure the standing balance using Footscan. It is a tool that can quantitatively measure the pressure delivered to the foot through a 1m long plantar force measuring plate that consists of 8192 pressure sensors (Fig. 3). Footscan examinations were conducted 3, 4, 8, 12, and 24th weeks after injury. In each session, the examinations were performed 3 times and the result were averaged. To confirm the changes in standing balance, we measured the travel distance for the center of pressure (COP) and the weight distribution of 4 quadrant areas: right, left, anterior, and posterior (Fig. 4).

**Figure 5.**
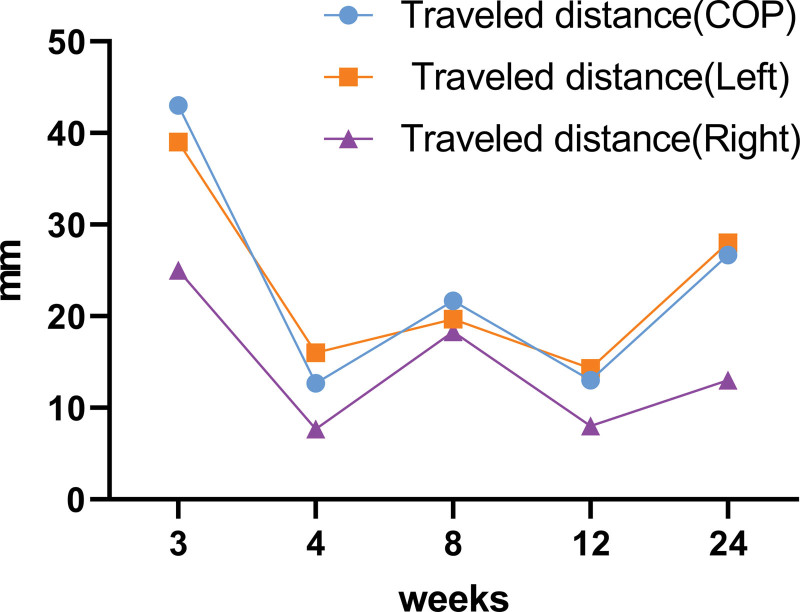
Time dependent change of travel distance on Footscan.

**Figure 6.**
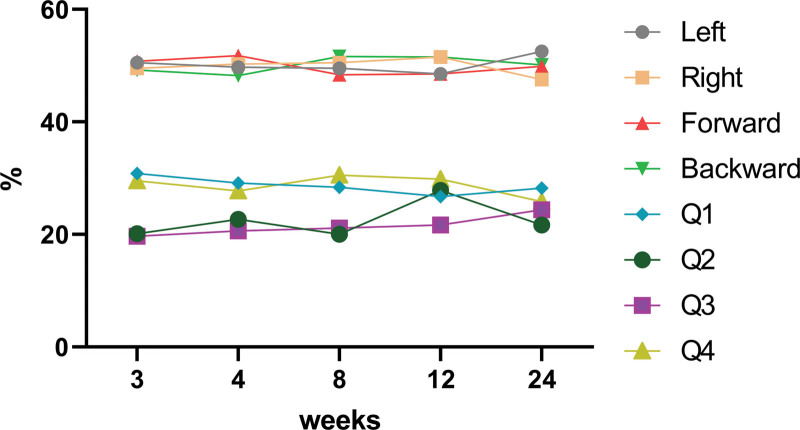
Time dependent change of weight distribution on Footscan.

The travel distance of the COP was longest in the 3rd week and decreased thereafter (Fig. 5). The weight distribution in the 4 quadrants remained similar without significant variability (Fig. 6).

## 3. Discussion

This case study describes quantitative changes in static standing balance in patients after LAS. Balance parameters were obtained using Footscan. The main indicator of static balance was the travel distance of the COP. In addition, the weight distributions of the 4 quadrants were obtained. In our study, there was improvement in static balance over time. The weight distribution between injured and uninjured feet did not differ significantly during the entire observation period.

Patients after LAS often show an antalgic gait pattern and develop poor balance towards the involved limb.^[14]^ The causes of such functional instability include balance deficit, joint position sense deficit, and delayed peroneal muscle reaction time.^[11]^ Previous studies have reported that the improvement of balance was obtained after improving proprioception of the affected ankle in patients with LAS. This means that a change in position sense for the ankle joint occurred after LAS.^[15–17]^ In addition, after short-term pain and impaired balance, subsequent chronic ankle instability or osteoarthritis should be considered. Therefore, examination and follow-up of balance impairments, caused by neuromuscular deficits are essential.

Among the previous studies, there have been many studies on how balance after LAS changes. One study used balance parameters such as postural sway (deviation from the COP)^[18]^ or the Rhomberg test.^[19]^ Most of these studies evaluated immediately after injury, making it difficult to determine changes over time. Furthermore, there were some limitations that these kind of parameters were insufficient to be objective or quantitative. Few studies have been conducted over the years. In this respect, we used Footscan to confirm changes in static balance for half a year to overcome these limitations.

This case report is meaningful in that it carried out a serial follow-up evaluation, up to approximately 6 months after the injury. The limitation of this study is that there was no pre-injury test, so we do not know the baseline condition. Future studies with multiple subjects are needed to confirm this relationship. From this study, we learned about the usefulness of Footscan as a means of monitoring balance changes after LAS.

## 4. Conclusion

A 36-year-old man visited our clinic with Grade II LAS. Over the following months, the patient showed reduced pain and could walk independently with improved static balance on the Footscan. In this study, we found that static balance, which reduced after LAS, was restored over time.

## Acknowledgements

The author received consent from the patient and thanks the authors for his consent to publish.

## Author contributions

**Conceptualization:** Kwangohk Jun, Won Mo Koo, Jeeihn Lee, Jong Min Kim, Byung Joo Lee, Tae-Woo Nam.

**Data curation:** Kwangohk Jun, Hyoshin Eo, Seongho Woo, Jeeihn Lee, Jong Min Kim, Byung Joo Lee, Tae-Woo Nam.

**Formal analysis:** Kwangohk Jun, Won Mo Koo, Jeeihn Lee, Jong Min Kim, Tae-Woo Nam.

**Investigation:** Kwangohk Jun, Seongho Woo, Jong Min Kim.

**Methodology:** Kwangohk Jun, Hyoshin Eo.

**Resources:** Won Mo Koo.

**Validation:** Kwangohk Jun.

**Visualization:** Kwangohk Jun.

**Writing – original draft:** Kwangohk Jun, Tae-Woo Nam.

**Writing – review & editing:** Kwangohk Jun, Byung Joo Lee, Tae-Woo Nam.

## References

[1] Woods C Hawkins R Hulse M. The football association medical research programme: an audit of injuries in professional football: an analysis of ankle sprains. Br J Sports Med. 2003;37:233–8. 12782548 10.1136/bjsm.37.3.233 PMC1724634

[2] Doherty C Delahunt E Caulfield B. The incidence and prevalence of ankle sprain injury: a systematic review and meta-analysis of prospective epidemiological studies. Sports Med. 2014;44:123–40. 24105612 10.1007/s40279-013-0102-5

[3] Fallat L Grimm DJ Saracco JA. Sprained ankle syndrome: prevalence and analysis of 639 acute injuries. J Foot Ankle Surg. 1998;37:280–5. 9710779 10.1016/s1067-2516(98)80063-x

[4] Bachmann LM Kolb E Koller MT. Accuracy of Ottawa ankle rules to exclude fractures of the ankle and mid-foot: systematic review. BMJ. 2003;326:417. 12595378 10.1136/bmj.326.7386.417 PMC149439

[5] Roos KG Kerr ZY Mauntel TC. The epidemiology of lateral ligament complex ankle sprains in national collegiate athletic association sports. Am J Sports Med. 2017;45:201–9. 27573356 10.1177/0363546516660980

[6] Gribble PA Bleakley CM Caulfield BM. Evidence review for the 2016 international ankle consortium consensus statement on the prevalence, impact and long-term consequences of lateral ankle sprains. Br J Sports Med. 2016;50:1496–505. 27259753 10.1136/bjsports-2016-096189

[7] Attenborough AS Hiller CE Smith RM. Chronic ankle instability in sporting populations. Sports Med. 2014;44:1545–56. 24981244 10.1007/s40279-014-0218-2

[8] Thompson C Schabrun S Romero R. Factors contributing to chronic ankle instability: a systematic review and meta-analysis of systematic reviews. Sports Med. 2018;48:189–205. 28887759 10.1007/s40279-017-0781-4

[9] Gross P Marti B. Risk of degenerative ankle joint disease in volleyball players: study of former elite athletes. Int J Sports Med. 1999;20:58–63. 10090465 10.1055/s-2007-971094

[10] Valderrabano V Hintermann B Horisberger M. Ligamentous posttraumatic ankle osteoarthritis. Am J Sports Med. 2006;34:612–20. 16303875 10.1177/0363546505281813

[11] Hertel J. Functional instability following lateral ankle sprain. Sports Med. 2000;29:361–71. 10840868 10.2165/00007256-200029050-00005

[12] Wolfe MW Uhl TL Mattacola CG. Management of ankle sprains. Am Fam Physician. 2001;63:93–104. 11195774

[13] Vuurberg G Hoorntje A Wink LM. Diagnosis, treatment and prevention of ankle sprains: update of an evidence-based clinical guideline. Br J Sports Med. 2018;52:956. 29514819 10.1136/bjsports-2017-098106

[14] Matherne T Cooke J McMorris M. Delayed conservative treatment of an acute lateral ankle sprain in a non-athlete female following walking boot immobilisation. BMJ Case Rep. 2019;12:e229625. 10.1136/bcr-2019-229625 PMC6663269 31352385

[15] Lephart SM Pincivero DM Giraldo JL. The role of proprioception in the management and rehabilitation of athletic injuries. Am J Sports Med. 1997;25:130–7. 9006708 10.1177/036354659702500126

[16] Lentell G Baas B Lopez D. The contributions of proprioceptive deficits, muscle function, and anatomic laxity to functional instability of the ankle. J Orthop Sports Phys Ther. 1995;21:206–15. 7773272 10.2519/jospt.1995.21.4.206

[17] Webster KA Gribble PA. Functional rehabilitation interventions for chronic ankle instability: a systematic review. J Sport Rehabil. 2010;19:98–114. 20231748 10.1123/jsr.19.1.98

[18] Guskiewicz KM Perrin DH. Research and clinical applications of assessing balance. J Sport Rehabil. 1996;5:45–63.

[19] Freeman MAR Dean MRE Hanham IWF. The etiology and prevention of functional instability of the foot. J Bone Joint Surg Br. 1965;47-B:678–85. 5846767

